# Impact of high drinking water nitrate levels on the endogenous formation of apparent *N*-nitroso compounds in combination with meat intake in healthy volunteers

**DOI:** 10.1186/s12940-019-0525-z

**Published:** 2019-10-17

**Authors:** Simone G. van Breda, Karen Mathijs, Virág Sági-Kiss, Gunter G. Kuhnle, Ben van der Veer, Rena R. Jones, Rashmi Sinha, Mary H. Ward, Theo M. de Kok

**Affiliations:** 1 Department of Toxicogenomics, GROW-school for Oncology and Developmental Biology, Maastricht University Medical Center, P.O Box 616, 6200 MD Maastricht, the Netherlands; 2 Department of Food & Nutritional Sciences, University of Reading, Reading, UK; 3 Occupational and Environmental Epidemiology Branch, Division of Cancer Epidemiology and Genetics, National Cancer Institute, National Institutes of Health, Bethesda, MD USA; 4 Metabolic Epidemiology Branch, Division of Cancer Epidemiology and Genetics, National Cancer Institute, National Institutes of Health, Bethesda, MD USA

**Keywords:** Nitrate, Nitrite, Drinking water, Processed red and unprocessed white meat, Human dietary intervention study, *N*-nitroso compounds, Genotoxicity, Vitamin C, Endogenous nitrosation

## Abstract

**Background:**

Nitrate is converted to nitrite in the human body and subsequently can react with amines and amides in the gastrointestinal tract to form *N*-nitroso compounds (NOCs), which are known to be carcinogenic in animals. Humans can be exposed to nitrate via consumption of drinking water and diet, especially green leafy vegetables and cured meat. The contribution of nitrate from drinking water in combination with meat intake has not been investigated thoroughly. Therefore, in the present pilot study, we examined the effect of nitrate from drinking water, and its interaction with the consumption of white and processed red meat, on the endogenous formation of NOCs, taking into account the intake of vitamin C, a nitrosation inhibitor.

**Methods:**

Twenty healthy subjects were randomly assigned to two groups consuming either 3.75 g/kg body weight (maximum 300 g per day) processed red meat or unprocessed white meat per day for two weeks. Drinking water nitrate levels were kept low during the first week (< 1.5 mg/L), whereas in week 2, nitrate levels in drinking water were adjusted to the acceptable daily intake level of 3.7 mg/kg bodyweight. At baseline, after 1 and 2 weeks, faeces and 24 h urine samples were collected for analyses of nitrate, apparent total *N*-nitroso compounds (ATNC), compliance markers, and genotoxic potential in human colonic Caco-2 cells.

**Results:**

Urinary nitrate excretion was significantly increased during the high drinking water nitrate period for both meat types. Furthermore, levels of compliance markers for meat intake were significantly increased in urine from subjects consuming processed red meat (i.e. 1-Methylhistidine levels), or unprocessed white meat (i.e. 3-Methylhistidine). ATNC levels significantly increased during the high drinking water nitrate period, which was more pronounced in the processed red meat group. Genotoxicity in Caco-2 cells exposed to faecal water resulted in increased genotoxicity after the interventions, but results were only significant in the low drinking water nitrate period in subjects consuming processed red meat. Furthermore, a positive correlation was found between the ratio of nitrate/vitamin C intake (including drinking water) and the level of ATNC in faecal water of subjects in the processed red meat group, but this was not statistically significant.

**Conclusions:**

Drinking water nitrate significantly contributed to the endogenous formation of NOC, independent of the meat type consumed. This implies that drinking water nitrate levels should be taken into account when evaluating the effect of meat consumption on endogenous formation of NOC.

**Trial registration:**

Dutch Trialregister: 29707. Registered 19th of October 2018. Retrospectively registered.

## Background

Nitrate is a naturally occurring compound in our environment that forms part of the nitrogen cycle. Plants absorb nitrate from the soil and ground water in order to obtain nitrogen, which is an essential component of plant proteins and chlorophyll [1]. Since the 1950s, the concentration of nitrate in our surroundings is rising, due to an increase in the release of nitrogen in the environment by human activity. Major contributors are fertilizers, animal and human waste products, and atmospheric deposition of nitrogen oxides from power plants and vehicle exhaust [2]. Nitrate which is not taken up by plants or which does not undergo denitrification will end up in groundwater and eventually in public drinking water supplies. Although exposure to high levels of nitrate in humans is mainly the result from consumption of nitrate-rich plants such as certain dark-green, leafy and root vegetables, consumption of contaminated drinking water may contribute substantially to total nitrate intake [2–4]. In specific regions in the world, e.g. in rural parts in India and the Gaza Strip, nitrate concentrations in drinking water are relatively high, and reach levels exceeding 100 mg/L [4].

Although nitrate in itself is not a carcinogen, exposure to high nitrate levels may have a genotoxic risk for humans due to the conversion of nitrate into nitrite by the oral microbiome [5, 6]. Nitrite can react with *N*-nitroso compound (NOC) precursors in the gastrointestinal tract, mainly amines and amides, thereby subsequently forming potentially carcinogenic NOCs [2, 3, 7–9]. Nitrite can also be present in low amounts in drinking water but is typically found in food items such as processed red meat products, where it is added to control pathogenic microbes, and prevent rancidity. Red and processed red meat also contain haem iron, which can act as a catalyst in the formation of NOCs, thereby contributing to increased exposure [10]. In addition, processed red meat products may contain low levels of pre-formed NOCs [11], which may further contribute to cancer development in humans with high dietary intake of meat.

As vegetables possessing high levels of nitrate also contain phytochemicals such as polyphenols and vitamin C, which are known to inhibit the process of endogenous nitrosation [9], intake of nitrate via drinking water may stimulate the formation of NOCs stronger as compared to nitrate intake through dietary consumption. Particularly the combination of high drinking water nitrate and processed red meat consumption, the latter of which stimulates nitrosation [7, 10], may result in increased exposure of the large intestine to NOCs and thereby increase colorectal cancer (CRC) risk. Although the relationship between intake of processed red meat and the increased risk of CRC is convincing according to both the Word Cancer Research Fund [12–14] and the International Agency for Research on Cancer (IARC) [15], the contribution of drinking water nitrate to the endogenous formation of NOCs and the subsequent increased risk of CRC has not been investigated thoroughly [2, 4, 16].

A number of epidemiological studies have investigated the relationship between drinking water nitrate levels and risk of CRC [17–21]. Positive associations have been found at drinking water nitrate concentrations below the current drinking water standard [21], for particular subgroups, e.g. subgroups with specific other dietary characteristics such as high meat intake [18], in combination with low vitamin C intake [17], or for subgroups with CRC related to a specific part of the colon [19].

A limited number of human biomonitoring studies have investigated the association between drinking water nitrate levels and generation of NOCs in the human body. Most of these studies report increased formation of endogenous NOCs after consumption of high drinking water nitrate (reviewed by Shamsuddin et al. [22]). For instance, Vermeer et al. showed that healthy female volunteers who consumed well water with high nitrate levels had higher levels of carcinogenic NOCs in their urine, which was associated with increased *HPRT* (hypoxanthine-guanine phosphoribosyltransferase) variant frequencies in lymphocytes [23]. This group also demonstrated that ingestion of nitrate in drinking water at the acceptable daily intake level of 3.7 mg/kg body weight in combination with a fish meal containing nitrosatable precursors increased the excretion of NOCs in urine of 25 healthy volunteers [24]. In a follow up study, the effect of the presence of nitrosation inhibitors in the diet on NOC excretion in urine was investigated. Results showed a decrease in the excretion of NOC in urine after simultaneous ingestion of vitamin C or moderate consumption of green tea, in combination with the fish diet and high level drinking water nitrate [25]. The presence of nitrosation inhibitors in the diet could be one of the reasons why epidemiological studies often fail to find a clear association between nitrate from drinking water and diet and cancer risk. More research is needed which investigates the role of NOC precursors and inhibiters in the diet after dietary nitrate intake in humans.

In this pilot study among healthy volunteers, subjects were randomly assigned to two groups consuming processed red meat or unprocessed white meat per day for two weeks. Drinking water nitrate levels were kept low (< 1.5 mg/L) during the first week, whereas in week 2, nitrate levels in drinking water were adjusted to the acceptable daily intake level. We investigated the effect of nitrate intake from drinking water, and its interaction with white and processed red meat, on the endogenous formation of NOCs and the genotoxic potential of faecal water. Furthermore, the impact of vitamin C intake, assessed by means of food diaries, on the formation of NOC was taken into account.

## Methods

### Subjects and study design

This pilot study was conducted in the context of the larger human dietary intervention study of the EU co-funded research study PHYTOME (www.phytome.eu), and included healthy volunteers above 18 years, with a normal weight BMI (18 kg/m^2^–25 kg/m^2^) recruited from the Faculty of Health Medicine and Life Sciences, Maastricht University, the Netherlands. Volunteers reported no problems or diseases of the gut, liver, kidney, heart or lungs including acute infections. All participants gave informed consent and the protocol was approved by the Ethics Review Committee of the Maastricht University Medical Centre (Registration number NL43956.068.13).

**Fig. 1:**
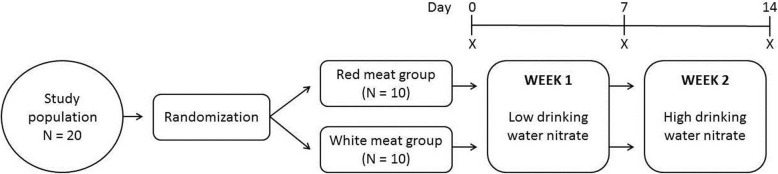
Study design. X = time point for sample collection (urine, faeces)

In total, 20 volunteers were recruited and randomly assigned to two groups (unprocessed white meat vs processed red meat). The intervention study consisted of two intervention periods of 7 days each, as shown in Fig. 1. During the first intervention period, volunteers were asked to consume 2 L per day of bottled drinking water with low nitrate levels (< 1.5 mg/L) in combination with 3.75 g/kg body weight (with a maximum of 300 g/day, based on previous studies [26, 27]) processed red meat or unprocessed white meat per day. During the second intervention period, volunteers were requested to consume 2 L per day of bottled drinking water with high nitrate levels in combination with the same amount of processed red meat or unprocessed white meat per day. The provided drinking water nitrate levels were adjusted individually to the Acceptable Daily Intake level (ADI: 3.7 mg/kg bodyweight). Subjects were requested to consume the entire amount of 2 L of water, and were not allowed to drink any additional water.

Processed red meat consisted of a variety of cooked and dry-cured red meat including bacon, ham and sausages. Unprocessed white meat consisted of chicken and turkey breast (Meat Factory, Henri van de Bilt B.V, Beuningen, the Netherlands). Meats were commercially available and provided to the volunteers so they had a similar day-to-day meat diet. No other meat products or fish products were allowed to be consumed during the intervention period. Volunteers kept track of their entire food intake during the study through the use of a food diary. At the beginning of the study (baseline) and after each intervention period, volunteers collected a faecal sample and 24 h urine for analysis. Samples were kept at 4 °C in provided storage boxes until storage at − 20 °C in our laboratories. Consumption of tea, coffee and alcohol were not permitted for the duration of the study and also the use of antibiotics in the prior month and during the study was not allowed.

### Chemicals and special consumables

All solvents and chemicals were of analytical grade or better, and were obtained from Sigma Aldrich (Dorset, UK).

### Generation of faecal water samples

Faecal water samples were prepared from faecal material collected from all volunteers at 3 different time points, i.e. at the beginning and end of each intervention period. After manual homogenization of the faecal material, samples were stored at − 20 °C until use. A small portion of homogenized faecal material (± 10 g) was ultracentrifuged at 50,000×g for 2 h at 10 °C. The supernatant faecal water was divided into aliquots and stored at − 20 °C until use.

### Analyses of nitrate in urine

Urine samples were analysed for nitrate using a chemiluminescence method described elsewhere [28]. Briefly, samples and standards containing nitrate were first reduced to nitric oxide (NO), which was then quantified using a NO analyser (NOA Eco Physics chemiluminescence detector, model 88 et). To reduce nitrate to NO, samples were added to 0.05 mol/L vanadium (III) chloride in 1 M hydrochloric acid refluxing at 90 °C. Vanadium chloride solution and NaNO_2_ standards were prepared fresh daily. Standards and samples were injected by disposable plastic syringes and needles directly in triplicates (coefficient of variations < 1%), samples were diluted 1:10 or 1:20 if needed. Thawed urine samples were kept in dark on ice and analysed within 2 h. Helium gas (purity 99.996%) was used to mix the sample and transfer released NO to the detector. The system was calibrated in the beginning of each batch with a minimum of 5 different concentrations NaNO_2_ (2.44–78 μM). EDAQ Software expressed concentrations as nitrate equivalent concentrations (μM).

### Analyses of 1- and 3-Methylhistidine levels in urine

1- and 3- Methylhistidine concentrations were determined using a Quattro Ultima triple quadrupole mass spectrometer (Waters, Milford,MA/; Micromass, Altrincham, U.K.) combined with a Waters Acquity UPLC system (Waters, Milford, MA). Chromatographic separation was achieved less than 6 min using a mixed mode column (Primesep 200 - SIELC, 2.1 × 100 mm, 5 μm, 100A, Crawford Scientific). Column was maintained at 35 °C. The Methylhistidine isomers were eluted with 0.4% of Formic acid, 30% Acetonitrile (pH = 3) at a flow rate of 0.2 mL/min. Standards and samples were diluted 1:10 by 2 μM isotope labelled internal standard (Tau-Methyl-D3-L-Histidine) and 10 μL was injected via CTC PAL autosampler. Standards were prepared from 1 mM frozen stock solutions in water: 500, 250, 125, 62.5, 31.25, 15.63, 7.81 and 3.91 μM for 1- and 3-Methylhistidine separately. Dilution were performed in a 96 well microplate and kept at 4 °C during the analysis. Internal standards, mobile phase and water were measured for quality control reasons. Blanks were monitored for carry over and showed no evidence of carryover contamination. Isomers were identified based on their retention time compared to standards and quantified by the ratio of their MRM transition (170.3 > 123.9 (CE:12) for 1-MH and 170.3 > 125.9 (CE:12)) peak areas to the isotope labelled internal standard peak area compared to ratios of external standards curves. The following ion source parameters were used: capillary voltage 3.5 kV, cone voltage 35 V, source temperature 100 °C, desolvation temperature: 250 °C, entrance lens 5, exit lens 5. Data was acquired and processed by Masslynx (Waters).

### Determination of apparent total *N*-nitroso compounds (ATNC) in faecal water

NOCs were measured as apparent total *N*-nitroso compounds (ATNC). ATNC concentrations were determined using a chemiluminescence detector (CLD) [28]. Thawed faecal water samples were kept in the dark on ice and analysed as soon as possible and within 2 h. 100 μL of faecal water sample was treated briefly with preservation solution (0.1 M N-ethylmaleimide and 0.01 M DTPA) and then incubated with 50 g/L sulfamic acid for 1–5 min. Nitrite content forms a diazo complex with the sulfamic acid that is stable in tri-iodide, this step is necessary to differentiate the nitrite content from the ATNC content. The sample was directly injected to the purge vessel (60 °C) containing 10–15 ml reduction solution (11.11 g/L potassium iodide and 5.55 g/L iodine in 40 mL water and 140 mL glacial acetic acid). Preservation solution was added to preserve the nitrosation state of thiols by alkylating free thiol groups and scavenging metal ions, which can cause a release of NO from nitroso-thiols. Tri-iodide reduction solution releases NO from nitrite, nitrosothiols, nitrosamines, iron-nitrosylhemoglobin and nitrosohemoglobin. ATNC contribution to the total CLD signal was determined by subtracting the nitrite response from the total response. All samples and standards were measured in duplicates.

### Analyses of genotoxicity in faecal water (comet-assay for DNA breakage)

The human colon adenocarcinoma cell line Caco-2 was used to test faecal water genotoxicity in the standard and formamidopyrimidine–DNA glycosylase (Fpg) comet assay as described by Singh et al. (1988) [29] and Pflaum et al. (1997) [30] with minor modifications. Fpg cuts the DNA strand specifically at oxidized purines and thus creates more strand breaks which represent oxidative DNA damage. Caco-2 cells (passage number 15–21) were cultured in DMEM (Sigma–Aldrich, Zwijndrecht, The Netherlands) supplemented with 1% (v/v) nonessential amino acids, 1% Na-pyruvate, 1% penicillin/streptomycin, and 10% (v/v) heat-inactivated foetal calf serum, all purchased from Gibco BRL (Breda, The Netherlands) and were incubated at 37 °C in a humidified incubator containing 5% CO_2_. The cells were harvested by trypsinization, centrifuged for 5 min at 200×g and re-suspended and incubated in growth medium containing 10% faecal water for 30 min incubation at 37 °C. After incubation, a small aliquot of cells (100 μL) were centrifuged (100×g, 3 min), re-suspended in low melting point agarose dissolved in phosphate-buffered saline and applied to the prepared slides.

Comets were visualized using a Zeiss Axioskop fluorescence microscope (at 200× magnification). Randomly, 50 cells were analysed using the Comet assay III software (Perceptive Instruments, Haverhill, UK). DNA damage was expressed as mean tail intensity (TI Percent DNA in the Tail). In each experiment, H_2_O_2_ exposed Caco-2 cells (100 μM, 30 min) were used as a positive control and were co-electrophorized and scored along with the faecal water-exposed cells to compensate for any inter-electrophoresis variation. Results are presented as mean ± standard error of the mean tail intensity relative to baseline.

### Analyses of food intake by means of a food diary

Participants were instructed to record their daily dietary intake during the study using an online standardized food diary from “Voedingscentrum” (https://mijn.voedingscentrum.nl) using the software program “Eetmeter” designed by the Netherlands Nutrition Center. For each food item, the amount consumed (standard portions: number of units, glasses, cups) was recorded per day. Food diaries were processed to calculate the average daily amounts of energy and nutrients using the “Eetmeter” database. Daily nitrite and nitrate intake were estimated using values from the published literature as described in Inoue-Choi et al. (2015) [31]. Nitrate intake from the food diaries was summed with the nitrate intake from drinking water to compute the total nitrate intake.

### Statistical analysis

Results of the data are expressed as mean ± standard error of the mean. Statistical analyses were conducted using two-sided t-tests to compare means for dietary nitrate and nitrite intake, urinary nitrate, faecal ATNC, and Comet assay results for the low and high drinking water nitrate periods. Paired sample t-tests were used when comparing means within individuals (i.e. low versus high drinking water nitrate). Independent t-tests were used to compare the processed red meat and unprocessed white meat groups.

For each subject, a ratio was calculated between dietary nitrate (including drinking water) and vitamin C intake, resulting in an index of the probability of formation of NOCs, as nitrate intake could increase the formation of NOCs and vitamin C could inhibit this process.

Linear regression analyses were used to examine relationships between nitrate intake and nitrate excretion in urine, and relationships between nitrate/nitrite intake and vitamin C intakes and ATNC. The threshold for significance in all analyses was set at *p* < 0.05.

## Results

### Study population

Nineteen participants (11 men, 8 women) completed the intervention study (see Table 1 for details). One participant (male) dropped out after the first week, due to influenza. There were no significant differences between the processed red meat group and unprocessed white meat group at baseline in regard to subject characteristics and excretion of urinary nitrate or faecal ATNC.

**Table 1:** Baseline characteristics of study participants

	Unprocessed white meat	Processed red meat
N	10	9
Age [year]	30 (3.9)	26.3 (2.5)
Sex (female)	5 (50%)	3 (33%)
Body weight [kg]	67.5 (3.3)	70.2 (4.1)
Current smoker	2 (20%)	0 (0%)
Urinary 1-Methylhistidine [μmol/day]	25.1 (7.0)	20.5 (3.5)
Urinary 3-Methylhistidine [μmol/d]	63.9 (36.9)	73.4 (23.0)
Urinary NO_3_^−^ [μmol/day]	740 (218)	715 (110)
Faecal water ATNC^†^[μmol/L]	15.8 (3.2)	16.7 (3)

Data are shown as mean (SEM: Standard error of the mean) or proportion

A statistically significant increase in compliance markers for intake of both meat types was observed. In subjects consuming unprocessed white meat, 3-Methylhistidine levels in urine were increased as compared to baseline (256 ± 50.9 and 296.8 ± 98.4 versus 63.9 ± 36.9 μmol/day), whereas a significant decrease was found in 3-Methylhistidine levels in urine from subjects consuming processed red meat and drinking water containing high nitrate levels (11.8 ± 2.5 versus 73.4 ± 23.0 μmol/day). The latter could be explained by the absence of consumption of white meat for two weeks, which could lead to this lower level of 3-Methylhistidine levels in the urine of the subjects. Furthermore, 1-Methylhistidine levels were significantly increased in subjects consuming processed red meat (29.1 ± 7.0 and 31.2 ± 5.5 versus 20.7 ± 3.5 μmol/day).

### Dietary intake of energy, macro- and micronutrients, and nitrite and nitrate levels

An overview of mean daily intakes of energy, macro- and micronutrients, and nitrite and nitrate for the processed red meat and unprocessed white meat group at baseline and during the low and high nitrate drinking water periods is shown in Table 2. During the low nitrate drinking water period, mean daily dietary nitrate intake (including drinking water nitrate) was similar in both meat groups and increased significantly with the consumption of high-nitrate drinking water (*p* < 0.001; 244 ± 15.8 versus 36.0 ± 3.4 mg/day in the unprocessed white meat group; and 255 ± 17.9 versus 53.8 mg/day in the processed red meat group). No other differences in intake of nutrients were observed within the meat groups during either the low or high drinking water nitrate periods. Comparing mean daily dietary intake between the two meat groups, intake of nitrite was higher in the processed red meat group than in the unprocessed white meat group (*p* < 0.001; 2.4 ± 0.1 versus 1.0 ± 0.1 mg/day). Furthermore, intake of fat (81.7 ± 4.1 versus 67.9 ± 3.2 g/day), zinc (11.6 ± 0.6 versus 9.0 ± 0.8 mg/day, vitamin D (2.8 ± 0.2 versus 1.9 ± 0.3 μg/day) (*p* < 0.05), sodium (5813.1 ± 329.3 versus 3202.6 ± 276.0 mg/day), and vitamin B1 (2.0 ± 0.1 versus 0.8 ± 0.1 mg/day (*p* < 0.01) was significantly higher, and intake of selenium (55.9 ± 2.5 versus 65.8 ± 1.9 μg/day (which is normally present in relative high amounts in red meat [32], but has also been reported to be present in high amounts in unprocessed white meat [33]), nicotinic acid (24.6 ± 1.3 versus 34.7 ± 1.3 mg/day, and vitamin B6 (1.8 ± 0.1 versus 2.5 ± 0.0 mg/day (*p* < 0.01) was significantly lower in the processed red meat group compared to the intake in the unprocessed white meat group.

**Table 2:** Mean (SEM) daily dietary intake of energy, macronutrients and micronutrients in the processed red and unprocessed white meat group during the low and high drinking water periods

Daily dietary intake Mean (standard error of the mean)	Unprocessed white meat group			Processed red meat group		
	Overall^a^	Low NO_3_ drinking water levels (< 1.5 mg/L)	High NO_3_ drinking water levels (ADI-levels)	Overall^a^	Low NO_3_ drinking water levels (< 1.5 mg/L)	High NO_3_ drinking water levels (ADI-levels)
Energy (kcal)	1927.7 (109.1)	1932.6 (104.0)	1928.9 (126.0)	2154.3 (69.2)	2119.0 (85.4)	2191.8 (72.9)
Fat (g)	67.9 (3.2)	70.8 (3.1)	65.5 (4.2)	81.7 (4.1) *	81.4 (5.5)	82.0 (4.7) *
Saturated fat (g)	26.5 (1.3)	28.2 (1.2)	25.0 (1.6)	31.7 (2.3)	31.3 (2.8)	32.2 (2.6) *
Carbohydrates (g)	194.4 (18.6)	189.7 (17.4)	199.5 (20.6)	214.3 (18.1)	209.0 (18.9)	219.9 (17.8)
Protein (g)	121.7 (3.9)	120.6 (4.5)	123.2 (4.7)	124.6 (21.4)	121.4 (20.0)	128.0 (23.0)
Fibers (g)	18.6 (2.0)	18.4 (1.9)	18.9 (2.2)	24.5 (2.6)	23.7 (2.3)	25.2 (3.0)
Nitrate (mg)	140 (35.5)	36.0 (3.4)	244 (15.8) ^###^	154.2 (36.9)	53.8 (7.5)	255 (17.9) ^###^
Nitrite (mg)	1.0 (0.1)	1.0 (0.1)	1.1 (0.1)	2.4 (0.1) ***	2.5 (0.1) ***	2.2 (0.2) ***
Sodium (mg)	3202.6 (276.0)	3152.5 (348.7)	3029.6 (325.0)	5813.1 (329.3) **	5500.7 (203.3) **	6130.2 (496.5) **
Potassium (mg)	3136.4 (261.4)	3131.8 (252.9)	3171.4 (289.3)	3179.4 (199.6)	3235.8 (180.9)	3124.2 (233.4)
Calcium (mg)	693.7 (98.8)	707.9 (104.0)	907.0 (236.7)	706.6 (66.2)	696.6 (85.2)	719.1 (61.7)
Magnesium (g)	332.5 (29.5)	330.0 (29.7)	364.2 (37.9)	332.1 (23.3)	328.2 (21.4)	336.3 (26.1)
Iron (mg)	10.7 (1.4)	11.0 (1.5)	37.4 (26.4)	11.2 (0.9)	11.1 (0.7)	11.3 (1.2)
Selenium (μg)	65.8 (1.9)	66.2 (3.1)	65.8 (2.1)	55.9 (2.5) **	56.4 (2.9) *	55.3 (3.2) *
Zinc (mg)	9.0 (0.8)	9.1 (0.9)	8.8 (0.8)	11.6 (0.6) *	11.4 (0.5)	11.8 (0.8) *
Vitamin A (μg)	464.9 (40.0)	485.8 (41.2)	446.3 (46.6)	398.6 (38.0)	388.6 (31.6)	409.0 (55.2)
Vitamin D (μg)	1.9 (0.3)	1.9 (0.3)	2.0 (0.3)	2.8 (0.2) *	2.7 (0.3)	2.9 (0.2) *
Vitamin E (mg)	9.6 (0.5)	9.5 (0.6)	9.7 (0.6)	8.1 (0.9)	7.8 (0.9)	8.5 (1.1)
Vitamin B1 (mg)	0.8 (0.1)	0.8 (0.1)	0.8 (0.1)	2.0 (0.1) **	2.0 (0.1) **	2.0 (0.1) **
Vitamin B2 (mg)	1.3 (0.2)	1.3 (0.2)	1.3 (0.2)	1.3 (0.1)	1.3 (0.1)	1.3 (0.1)
Vitamin B6 (mg)	2.5 (0.0)	2.4 (0.1)	2.5 (0.1)	1.8 (0.1) **	1.8 (0.1) **	1.8 (0.1) **
Folic acid (μg)	225.6 (49.0)	228.1 (49.2)	223.4 (49.2)	213.2 (14.3)	218.4 (16.9)	208.2 (13.4)
Vitamin B12 (μg)	3.8 (0.6)	3.7 (0.6)	3.9 (0.6)	3.3 (0.2)	3.3 (0.3)	3.3 (0.3)
Nicotinic acid (mg)	34.7 (1.3)	35.0 (1.6)	34.5 (1.4)	24.6 (1.3) **	24.6 (1.0) **	24.6 (1.8) **
Vitamin C (mg)	57.8 (7.9)	54.6 (8.0)	61.3 (11.0)	75.9 (10.2)	77.5 (11.4)	74.4 (10.3)

^a^overall: data combined for low and high drinking water period;

*p < 0.05;**p < 0.01, ***p < 0.001; Independent t-test processed red meat group vs unprocessed white meat group;

^###^*p* < 0.001; Paired samples t-tests comparing means between individuals (i.e. low versus high drinking water nitrate)

### Analyses of exposure markers in urine and faecal water

**Fig. 2:**
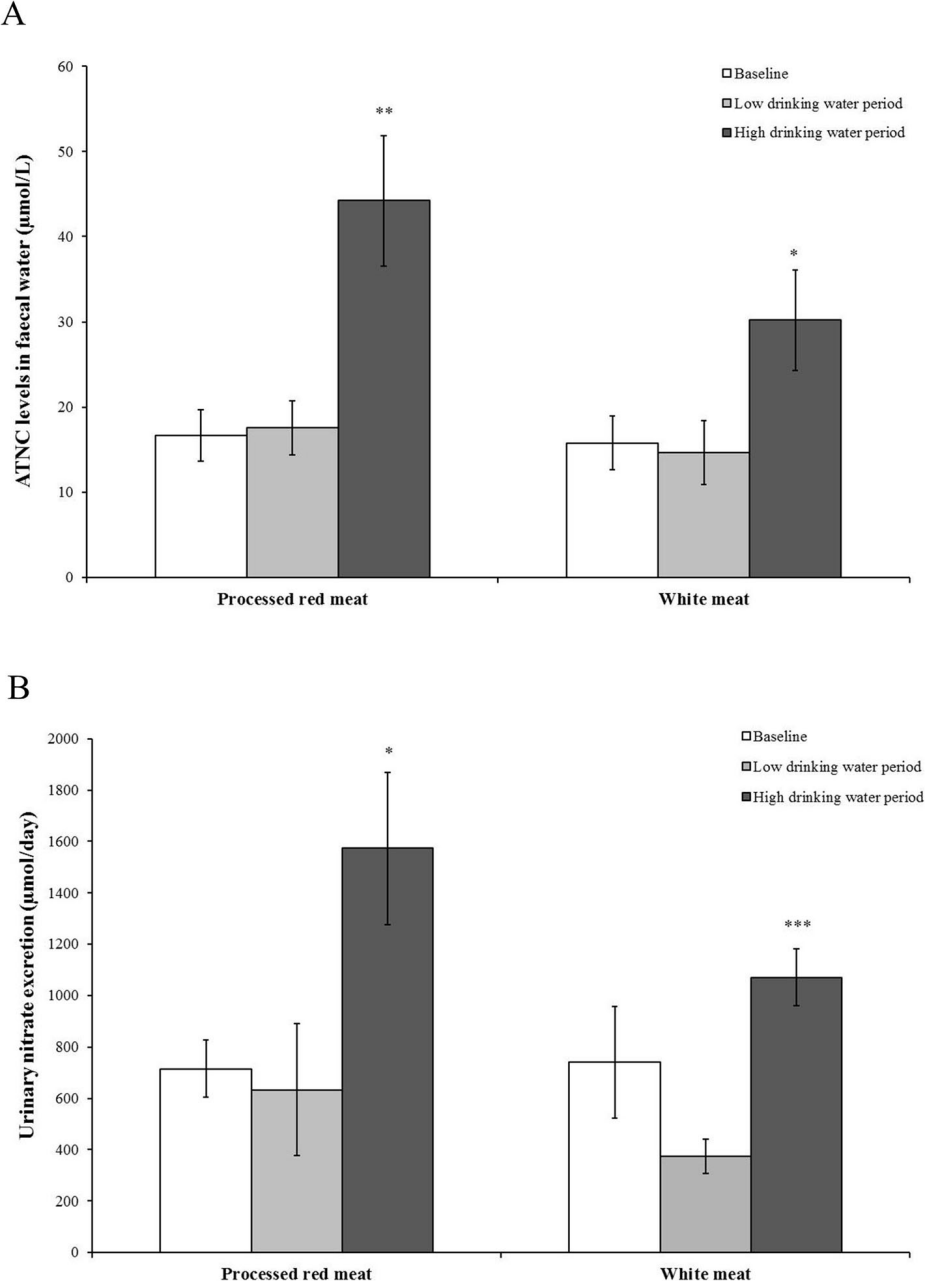
**a** ATNC levels in faecal water (Mean ± standard error of the mean (SEM); μmol/L) at baseline, after the low drinking water (< 1.5 mg/L) and after the high drinking water (ADI levels) period for the processed red meat group and unprocessed white meat group. ATNC levels and urinary nitrate excretion significantly increased after the high drinking water period in both the processed red meat group and unprocessed white meat group (** *p* < 0.01, * *p* < 0.05, respectively); **b** Nitrate levels in urine (Mean ± SEM; μmol/day) at baseline, after the low drinking water and after the high drinking water period for the processed red meat group and unprocessed white meat group. Urinary nitrate excretion significantly increased after the high drinking water period in both the processed red meat group and unprocessed white meat group (* *p* < 0.05, *** *p* < 0.001, respectively)

There were no statistically significant differences in faecal water ATNC levels and urinary nitrate excretion between the processed red meat group and the unprocessed white meat group at baseline and during the low drinking water period; however, ATNC levels and excretion of urinary nitrate increased significantly following the high drinking water nitrate period (Fig. 2a and b, respectively, as compared to the low drinking water nitrate period; *p* < 0.01 (44.2 ± 7.7 versus 17.6 ± 3.2 μmol/L) and *p* < 0.05 (30.2 ± 6.0 versus 14.7 ± 3.8 μmol/L) for ATNC levels for processed red and unprocessed white meat, respectively; *p* < 0.05 (1572 ± 295 versus 634 ± 255 μmol/day) and *p* < 0.001 (1071 ± 111 versus 375 ± 67 μmol/day) for urinary nitrate excretion for processed red and unprocessed white meat, respectively). The difference in faecal ATNC levels between the low and high drinking water period was more pronounced in participants consuming the processed red meat (mean difference 26.6 μM, *p* < 0.01) compared to participants consuming the unprocessed white meat (mean difference 15.5 μM, *p* < 0.05) (Table 3).

**Table 3:** Mean (standard error of the mean)) of urinary nitrate, 1-Methylhistidine, and 3-Methylhistidineexcretion, faecal water apparent nitroso compounds (ATNC) and Comet assay tail intensity levels for the unprocessed white and processed red meat group at baseline and after the low and high-nitrate (NO_3_^−^) drinking water periods

	Unprocessed white Meat				Processed red Meat			
	Baseline	Low NO_3_^−^ drinking water levels (< 1.5 mg/L)	High NO_3_^−^ drinking water levels (ADI- levels)	p_t-test_	Baseline	Low NO_3_^−^ drinking water levels (< 1.5 mg/L)	High NO_3_^−^ drinking water levels (ADI-levels)	p_t-test_
Urinary NO_3_^−^ [μmol/day]	740 (218)	375 (67)	1071 (111)	< 0.001^a^ 0.19^b^ 0.13^c^	714 (110)	634 (255)	1572 (295)	< 0.05^a^ 0.05^b^ 0.78^c^
Urinary 1-Methylhistidine [μmol/day]	25.1 (7.0)	15.8 (3.3)	18.6 (5.0)	0.64^a^ 0.46^b^ 0.25^c^	20.7 (3.5)	29.1 (7.0)	31.2 (5.5)	0.81^a^ < 0.05^b^ 0.30^c^
Urinary 3-Methylhistidine [μmol/day]	63.9 (36.9)	256.6 (50.9)	296.8 (98.4)	0.72^a^ < 0.01^b,c^	73.4 (23.0)	26.2 (11.3)	11.8 (2.5)	0.24^a^ < 0.05^b^ 0.09^c^
Faecal water ATNC^†^ [μmol/L]	15.8 (3.2)	14.7 (3.8)	30.2 (6.0)	< 0.05^a^ 0.05^b^ 0.83^c^	16.7 (3.0)	17.6 (3.2)	44.2 (7.7)	< 0.01^a,b^ 0.84^c^
Comet^d^	100.0 (0.0)	157.3 (37.9)	152.9 (50.4)	0.89^a^ 0.35^b^ 0.16^c^	100.0 (0.0)	173.2 (28.7)	138.0 (23.4)	0.35^a^ 0.21^b^ < 0.05^c^

^a^) difference between low NO_3_^−^ and high NO_3_^−^ drinking water period; ^b^) difference between high NO_3_^−^ drinking water period and baseline; ^c^) difference between low NO_3_^−^ drinking water period and baseline

^d^tail intensity levels relative to baseline

### Analyses of genotoxicity in faecal water (comet-assay for DNA breakage)

No statistically significant differences in faecal water genotoxicity were found between the high and low drinking water nitrate periods in both the processed red meat group and the unprocessed white meat group. Only after the low drinking water nitrate period, DNA damage was significantly higher in the processed red meat group compared to baseline levels (*p* < 0.05; 173.2 ± 28.7%) (Table 3).

### Association between exposure markers, effect markers and diet

**Fig. 3:**
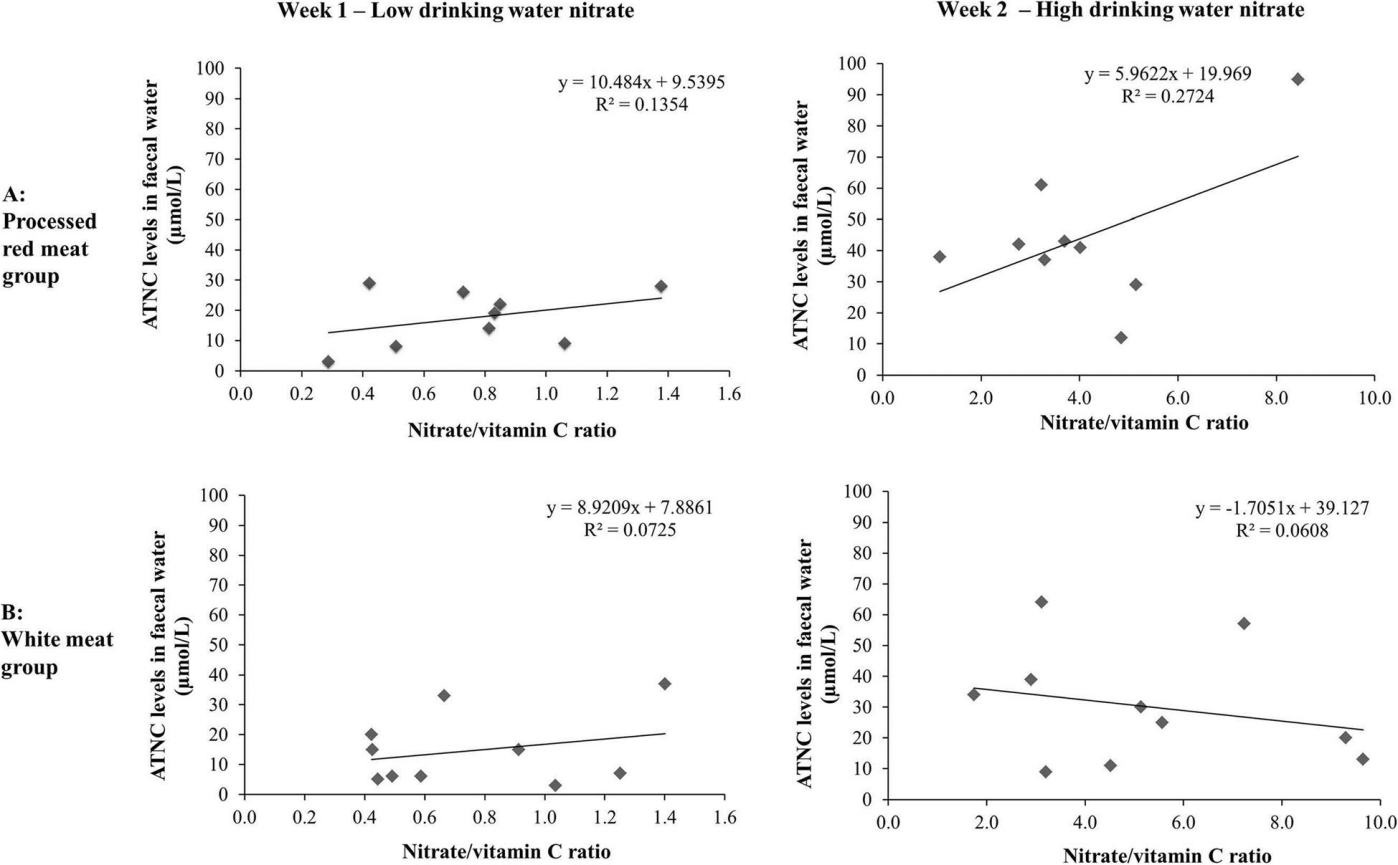
**a** Correlation between the ratio of nitrate and vitamin C intake and ATNC levels in faecal water for subjects in the processed red meat group at the low drinking water (< 1.5 mg/L) and at the high drinking water (ADI levels) period (R^2^ = 0.2724; *p* = 0.15); **b** Correlation between the ratio of nitrate and vitamin C intake and ATNC levels in faecal water for subjects in the unprocessed white meat group at the low drinking water and at the high drinking water period

Total urinary nitrate excretion was positively associated with total nitrate intake in the high drinking water period for all subjects (Spearman Rho = 0.46; *p* < 0.05). No significant associations were found between ATNC levels in faecal water and nitrate or nitrite intake in either meat groups. In the processed red meat group, a positive correlation was observed between ATNC levels in faecal water and the ratio of nitrate and vitamin C, but this was mainly driven by one subject and not statistically significant (R = 0.27, *p* = 0.15) (Fig. 3a).

## Discussion

The endogenous formation of NOCs is proposed as one of the key mechanisms underlying the positive association between colorectal cancer risk and processed meat consumption [32], or the intake of dietary nitrate and nitrite [3, 34]. However, the formation of endogenous NOCs is dependent on additional factors, like the presence of nitrosation precursors and haem iron which may stimulate their formation, or dietary ingredients that may act as nitrosation inhibitors such as vitamin C, vitamin E, and various polyphenols. Establishing the effect of dietary nitrate and nitrite on the nitrosation process is therefore problematic, as ingestion of particular nitrate and nitrite rich food products like green leafy vegetables also contain high amounts of a wide variety of nitrosation inhibitors.

This is the first human dietary intervention study investigating the effect of drinking water nitrate levels in combination with consumption of either processed red meat or unprocessed white meat on endogenous nitrosation and genotoxicity of faecal water in healthy volunteers. Genotoxicity of faecal water was increased after consumption of both processed red meat and unprocessed white meat, however, due to the high variation in the results, only the comparison between baseline and the processed red meat group in combination with low nitrate drinking water levels was statistically significant. This is an unexpected finding which cannot be explained biologically, and might be due to chance. Endogenous nitrosation was assessed by measurement of ATNC levels as measure of total NOC in faecal water. We show that, at relatively low drinking water nitrate levels, there is no statistically significant difference in faecal ATNC between baseline levels and levels after a 1 week intervention with either 3.75 g/kg body weight (maximum of 300 g/day) of processed red or unprocessed white meat per day. However, at high drinking water nitrate levels (ADI levels), ATNC levels were significantly increased. These results show that nitrate in drinking water had a significant contribution to the endogenous formation of ATNC, independent of the type of meat consumed. Notably, this difference in ATNC levels between the low and high drinking water period was more pronounced for the subjects consuming processed red meat than for those consuming unprocessed white meat. The ADI level which is used in this study comprises nitrate from dietary sources that includes nitrate from drinking water. The ADI levelis not directly related to the drinking water standard as the allowable intake varies by the person’s weight. However, the level of nitrate which is used in the drinking water exceeds the regulatory limit of 50 mg/L nitrate by the WHO.

The findings of our study are in line with a previous human dietary intervention study, showing increased excretion of NOCs in urine of subjects consuming drinking water with nitrate levels at ADI level in combination with a fish meal containing nitrosation precursors [24], and with results from a human dietary intervention study by Rowland et al. (1991) who demonstrated a significant increase in faecal ATNC concentrations in subjects consuming 300 mg nitrate/day in drinking water for 4 days [35].

In addition to considering the contribution of several nitrosation precursors in the overall assessment of cancer risk and nitrate intake, it is important to include the impact of nitrosation inhibitors. Taking into account dietary vitamin C intake in our study, we found a positive, although not statistically significant, association between endogenous ATNC-formation among subjects consuming relatively high levels of nitrate and low levels of vitamin C. However, this association was mainly driven by one person. Mirvish et al. have shown that the timing of vitamin C intake in combination with nitrosation precursors is of importance for inhibition of nitrosation [36–38]. As vitamin C intake was not administered in a controlled manner (dose and timing), but was assessed by means of food diaries, we could not establish a strong correlation between vitamin C intake, nitrate intake and NOC levels.. Furthermore, no statistically significant difference in mean vitamin C intake in the different study groups was observed. But this demonstrates that stable vitamin C intake in combination with elevated nitrate intake, could lead to increased NOC formation. These findings are in concordance with the already mentioned human dietary intervention study from Vermeer et al. (1998) on high drinking water nitrate levels in combination with a fish meal containing nitrosation precursors [24]. This study showed that simultaneous ingestion of nitrosation inhibitors like vitamin C or green tea was able to significantly decrease NOC levels in urine [25]. In a more recent dietary intervention study in obese men, the combined contribution of various dietary compounds on endogenous NOC formation was assessed [39]. Results showed that endogenous NOC formation is driven by increased red meat and nitrate intake, total energy levels, and reduced intake of vitamin C and non-starch polysaccharides. A negative association between vitamin C intake and a positive association between dietary nitrate intake and faecal NOC levels was found. Furthermore, this association became even stronger when analysing both nitrate and vitamin C intakes simultaneously (either as separate variables or as nitrate/vitamin C ratio). Intake of dietary nitrate ranged from moderate (80 mg/day) to high (443 mg/day) levels and was calculated based on food diaries.

In addition to these human biomonitoring studies, assessment of intake of NOC precursors from the diet and the incidence of colorectal cancer has been carried out in a limited number of epidemiological studies. Our data are supportive of observations from a recent case-control study in Spain and Italy, in which a positive association between drinking water nitrate levels (> 10 mg/day versus ≤5 mg/day) and CRC risk was found, in particular among subgroups with high red meat intake [18]. Average drinking water nitrate levels ranged from 3.4 to 19.7 mg/day, among the different areas, values which are below current international guidelines of 50 mg/L of the World Health Organization [40]. Some of the epidemiological studies take simultaneous intake of NOC inhibitors from the diet into account as well. In a case-control study conducted among residents in Iowa, negligible overall associations between colon and rectum cancers with measures of nitrate in public water supplies were found. However, increased risk of colon cancer was reported among subgroups exposed for more than 10 years to drinking water containing more than > 5 mg/L nitrate (as nitrogen; equivalent to 22 mg/L as NO_3_) and consuming lower levels of vitamin C or high amounts of red meat [17]. In addition, in the Shanghai Women’s Health study, an ongoing prospective cohort study of 73,118 women living in Shanghai, a higher risk of colorectal cancer was reported among women with vitamin C intake below the median (83.9 mg/day) and increasing quintiles of dietary nitrate intake [41].

Although our study is limited in number of subjects and the intervention periods are relatively short, we were able to demonstrate a significant increase in ATNC levels in faecal water of healthy humans consuming drinking water with high levels of nitrate. Furthermore, our results emphasize the importance of taking both nitrosation precursors as well as nitrosation inhibitors into account in the assessment of the nitrate intake on cancer risk.

## Summary and conclusions

Previous studies show an increased formation of endogenous NOC as well as an increased risk of CRC as a consequence of nitrate intake, even in populations consuming drinking water with nitrate levels below current guideline levels of 50 mg/L. In particular, subjects consuming low levels of vitamin C in combination with high levels of potentially harmful components like processed red meat and nitrate from drinking water may be at increased risk. The results of the current human dietary intervention study show that drinking water nitrate can have a significant contribution to the endogenous formation of NOCs, independent of meat type consumed. The effect is, however, more pronounced in subjects consuming processed red meat.Based on these suggestive findings and the classification of processed meat as carcinogenic by the IARC, risk assessments should also take into account drinking water nitrate levels.

## Data Availability

The datasets used and/or analysed during the current study are available from the corresponding author on reasonable request.

## Abbreviations

ADI: Acceptable daily intake
ATNC: Apparent total *N*-nitroso compounds
CLD: Chemiluminescence detector
CRC: Colorectal cancer
Fpg: Formamidopyrimidine–DNA glycosylase
HPRT: Hypoxanthine-guanine phosphoribosyltransferase
IARC: International agency for research on cancer
NO: Nitric oxide
NO_2_^−^: Nitrite
NO_3_^−^: Nitrate
NOC: *N*-nitroso compounds
SEM: Standard error of the mean
WHO: World Health Organization
